# The joint action MODE (Mutual Organ Donation and Transplantation Exchanges): a sound contribution to implementation of health policies in organ donation and transplantation

**DOI:** 10.1186/0778-7367-71-3

**Published:** 2013-02-19

**Authors:** Paola di Ciaccio, Claudia Ferraro, Pavel Brezovsky, Eduardo Martìn-Escobar, Alessandro Nanni Costa

**Affiliations:** 1 Centro Nazionale Trapianti, Istituto Superiore di Sanità, Rome, Italy; 2 Koordinacni Stredisko Transplantaci, Prague, Czech Republic; 3 Organización Nacional de Trasplantes, Madrid, Spain

## Abstract

**Background:**

The main objective of the joint action MODE is the transfer of best-practices in the field of organ donation and transplantation and the creation of positive synergies among participating European (EU) Member States (MS) apt to support authorities in possible decision-making and policy contexts.

**Methods:**

The consortium has chosen to foster the exchange of best-practice through a series of exchange visits followed by the provision of a set of specialized trainings.

Each participating MS has presented its strengths and weaknesses through a questionnaire based on the Organ Action Plan. Once the situation was clearer, countries with the strongest program organized and hosted the on-site visits and each country had the opportunity to perform five exchange visits on five selected topics.

Specific courses for healthcare staff of organ coordinating and transplantation centres were organized. Based on evaluation of the results of the on-site visits and training needs indicated by the partners, the chosen topics were:

• reporting on adverse events and reactions

• quality assurance programme of the donation process in Spain

• quality assurance of the transplantation process

**Results and conclusions:**

The outcome is that within the EU, even among MS with well-developed services, the organ donation and transplantation activity has substantial differences so that all participating countries would benefit from investigating foreign donation and transplant systems. Collaboration at EU level can be beneficial for all systems and the joint action MODE indicated that in some countries the sharing of expertise across the EU Member States has already proved to be useful in starting a virtuous circle in organization and training that would allow to increase organ donor rates and improve overall performance.

## Background

For many patients, organ transplantation represents the only lifesaving treatment available, a successful therapy for some categories of patients suffering from serious organ failures. This treatment has a positive outcome on the medium and long term for about 80% of the patients. This therapeutic opportunity is however precluded to a number of patients due to organ shortage. In 2003 the European Commission performed a survey [1] from which it emerged that 56,000 patients were waiting for a suitable organ donor in the EU and every day 12 people were dying while waiting for transplantation. Based on data collected yearly by ONT [2] (Organization Nacional des Transplantes) and published under the aegis of the Council of Europe, this amount totted up to about 47.700 waiting individuals in 2010. On the other side, cadaveric donation rates are more or less stagnant on an average, since average number of cadaveric donors per million of population was equal to 17.1 in 2008, 17.0 in 2009 and 16.9 in 2010, with great variations between EU Member States (MS). The European Commission has therefore committed to identify the major policy challenges in the field of organ donation and transplantation with priority toward (1) ensuring the quality and safety of human organs, (2) increasing organ availability and (3) enhancing the efficiency and accessibility of transplantation systems in the European Union. These three challenges are effectively addressed by the “Directive on standards of quality and safety of human organs intended for transplantation” (2010/53/EU) [3] adopted by the EU Parliament on July 7 2010 and by Action Plan on Organ Donation and Transplantation, aiming at strengthening cooperation between Member States. The Directive provides for the appointment of Competent Authorities in all Member States, for authorization of procurement and transplantation centres and activities, for traceability systems, as well as for the reporting of serious adverse events and reactions and EU MS should have implemented such requirements in their legal systems by 27 August 2012. As it is well indicated in the white paper issued in 2007 by the EU Commission “Together for Health: A strategic approach for the EU: 2008-2013” [4], MS have the main responsibility for health policy and provision of healthcare to European citizens but there are areas where MS cannot act alone effectively. Organ donation and transplantation is a great example of application of such strategy, that the whole transplant community hopes will prove to be successful in the near future.

## Methods

The Joint Action tool is the best methodological instrument suited to enable participating MS to further contribute to the process identified by the European Commission, since a formal commitment is required by Member States that delegate partners to perform the action. The Joint Action “Mutual Organ Donation and Transplantation Exchanges – MODE” is an 18-month project co-funded by the European Commission under the Public Health Program (DG Health and Consumer Protection), Grant 20102101. It started on January 1st 2011 and ended on June 30th 2012. This joint action was meant to contribute to the on-going EU policy allowing transmission of best practices in the three above-mentioned fields (quality and safety, organ donation, efficiency and accessibility of transplant systems) identified by the EU 53/2010 Directive as priority sectors of action. Table 1 describes the major criteria used for defining “best practices” in each of these fields.

**Table 1:** Criteria for definition of best practices in the mutual organ donation and transplantation exchanges, EU joint action MODE, 2011–2012

Quality and safety	Overview of quality/safety national programmes and evaluation of programme results, with special attention to programmes for notification of adverse events and reactions
Organ donation	Overview of national programmes for monitoring devastating brain injuries in ICUs and analysis of donation rates
Efficiency and accessibility of transplant systems	Analysis and comparison of existing national programmes for evaluation of transplant outcomes

The joint action Mode intended to complement MS policies in the field of organ donation and transplantation through cross-border cooperation. Such best-practice know-how transfer has been implemented through a series of actions starting with the identification of interest sectors, followed by exchange visits with a focus on identified items and specific training. The expected outcome was that since even European Union countries with well-developed services show significant differences in organ donation and transplantation activity, all participating countries would have benefited from investigating foreign donation and transplant systems in place. Another positive expected effect was the creation of positive synergies among participating MS apt to support authorities in possible decision-making and policy contexts.

## Results and Discussion

In the organ donation and transplantation field each country has its own history and practices, its own organization, structure and conditions, and something to offer its counterparts and some needs to be coped with. The Joint Action MODE consortium, that has been coordinated by Centro Nazionale Trapianti / Istituto Superiore di Sanità – Italy, involved as associated partners a good number of national organ donation and transplant organizations from several EU MS, namely, Országos Vérellátó Szolgálat – Hungary, Autoridiade para os Serviçios de Sangue e de Transplantação – Portugal, Organización Nacional de Trasplantes – Spain, Koordinacni Stredisko Transplantaci – Czech Republic, Slovenija Transplant – Slovenia, Pauls Stradins Clinical University Hospital – Latvia, Lithuanian National Transplantation Bureau Lithuania, Tartu University Hospital – Estonia, Bulgarian Executive Agency for Transplantation – Bulgaria and Mater Dei Hospital – Malta. The consortium partnership was therefore a good representative sample of MS from different EU areas and allowed to gather a number of different approaches to the organ donation and transplantation issue.

During the first months of the project, in order to identify the field of action for the exchange visits, a questionnaire based on the Organ Action Plan was circulated among partners in order to allow each country to analyze and present its strengths and weaknesses. In the same questionnaire a section was devoted to a survey on training needs. The partner responsible for such activity (KST from Czech Republic) worked out a chart flow with answers that was discussed by the consortium and a SWOT (Strenghts/Weaknesses/Opportunities/Threats) analysis was applied at national level, so that replies to the questionnaire were processed in a point diagram showing the score and level of intensity of individual replies.

As outcome of this SWAT analysis and upon final decision of the whole consortium, five topics and five countries were singled out and onsite visits were organized accordingly. Each country had the opportunity to take part in each of such visits. Table 2 gives an overview of the topics of the visit and location, together with reference to Organ Action Plan goal.

**Table 2:** Summary of on-site exchange visits

**Hosting partner**	**Organ action plan priority**	**Main topic**
ONT Spain	(1) Increasing organ availability	Quality improvement programs
KST Czech Rep.	(2) Enhancing efficiency and availability of transplant systems	Supporting and guiding organizational models
ASST Portugal	(2) Enhancing efficiency and availability of transplant systems	Supporting international exchange of organs for transplantations
ST Slovenia	(2) Enhancing efficiency and availability of transplant systems	Increasing deceased donation to their full potential
CNT Italy	(3) Improving quality and safety of transplants	Evaluation of post-transplantation results

At the end of the five visits, each partner wrote a memorandum explaining if the topic chosen could be implemented in his country and how this could be done while at the same time providing a description of barrier which may hamper the implantation. As a result, in October 2011, a final detailed report of such visits describing their outcome was drawn up.

On the basis of onsite visit outcomes and identified training needs, three topics were selected and training courses were developed by the responsible partner, ONT from Spain, in close collaboration with the whole consortium. The topics were the “Reporting on adverse events and reactions”, the “Quality assurance program of the donation process” and the “Quality assurance of the transplantation process”. The reporting of adverse events and reaction was a completely new topic and had not been covered by any specific exchange visit, but the growing interest for this activity and its focus on important aspects of the implementing Directive such as bio vigilance and surveillance on substances of human origin in Europe pushed the consortium to add specific training on this issue, whereas the other two courses were concentrated on quality indicators for donation processes and organization of registries and methodologies for analysis of transplant outcomes. Training courses included two phases, online face-to-face and were mainly addressed to the staff from national organizations or Competent Authorities that were directly working in the field of organ donation and transplantation. From March 1st to 31st, attendees were enrolled and they were provided with educational material. Next, they were assigned a user-id and password to access a webplatform through which they took part into an e-learning session from April 16th to 26th that included tests and surveys and forum discussions. Eventually, from May 7 to 9th three face-to-face courses (eight hours each) were held in Madrid at the Escuela Nacional de Sanidad (National School of Health). Content finalization is shown in Table 3. The courses have been certified by the MODE consortium.

**Table 3:** Content of the training for mutual organ donation and transplantation exchanges, EU joint action MODE, 2011–2012

**Issue**	**Topic**
Reporting on Adverse Event and Reactions	Fundamentals of risk management
	Overview. – Need for a safety system. The Italian approach
	Organization of bio vigilance system (by the Competent Authorities and Structure and responsibilities)
	Tools to support decisions: literature and Notify project
	Simulated exercises of bio vigilance
The Quality Assurance Program of the donation Process in Spain: Key Factors to improve	General description of the Spanish “Quality Assurance programme of the donation Process”
	The internal evaluation phase: objectives, methodology, tools and database, results and usefulness
	Practical work: quality indicators, results and analysis
	The external audit: key-points. Objectives, methodology, tools and database, results and usefulness
	Practical work: the external audit report: Practical cases
Quality Assurance of the Transplantation Process	Quality indicators for assessing organ transplant
	Analysis of outcomes
	Organisation of registries and collaboration with internal registries
	External audits of transplant centries

## Conclusions

From a general point of view the major action output was the acquired knowledge of best-practice system and also the awareness of the obstacles that hinder the full transfer of such practices in donation and transplant systems. Indeed, the fact that Directive implementation date was set for end August 2012 and that a number of countries are still discussing many details of such implementation did not allow us to make a full evaluation of long-term effects of our efforts, above all in measureable terms. During the last meeting held in Rome end June 2012, each partner was asked to deliver a presentation on the concrete possibility of best practices transfer, and on the state of the art of Directive implementation. They also illustrated which topics of the training they had found as most useful. That was the occasion for an open discussion of the results obtained through the joint action and on the obstacles mainly caused by the local political situation, e.g. lack of resources.

One of the main results emerged during the final meeting is that the importance of Authorities and policy makers involvement in order to obtain the needed resources and reach set goals. It is also worthwhile mentioning that all partners stressed that investment of resources and organizational efforts are more than ever needed in order to achieve desired results in organ donation and transplantation field, and unfortunately the present economic situation in Europe does not foster such approach. The main positive conclusion was no doubt the general agreement on the usefulness of having identified the fields where best practice transfer is most needed and of having given the possibility to train dedicated staff from Competent Authorities on such topics: a first step forward and a correct way of investing the resources allocated by the European Union to such action.

For further information and follow-up of our project, please visit the website “http://www.mode-ja.org/node/2”.

## Competing interests

The authors declare that they have no competing interests.

## Authors’ contributions

All the authors were workpackage leader, that implies they had a pivotal role in the project. All authors read and approved the final manuscript.
